# Use of the γ-H2AX Assay to Investigate DNA Repair Dynamics Following Multiple Radiation Exposures

**DOI:** 10.1371/journal.pone.0079541

**Published:** 2013-11-29

**Authors:** Luca G. Mariotti, Giacomo Pirovano, Kienan I. Savage, Mihaela Ghita, Andrea Ottolenghi, Kevin M. Prise, Giuseppe Schettino

**Affiliations:** 1 Dipartimento di Fisica, Università degli studi di Pavia, Pavia, Italy; 2 Centre for Cancer Research and Cell Biology, Queen’s University Belfast, Belfast, United Kingdom; 3 Istituto Nazionale di Fisica Nucleare, Sezione di Pavia, Pavia, Italy; Northern Institute for Cancer Research, United Kingdom

## Abstract

Radiation therapy is one of the most common and effective strategies used to treat cancer. The irradiation is usually performed with a fractionated scheme, where the dose required to kill tumour cells is given in several sessions, spaced by specific time intervals, to allow healthy tissue recovery. In this work, we examined the DNA repair dynamics of cells exposed to radiation delivered in fractions, by assessing the response of histone-2AX (H2AX) phosphorylation (γ-H2AX), a marker of DNA double strand breaks. γ-H2AX foci induction and disappearance were monitored following split dose irradiation experiments in which time interval between exposure and dose were varied. Experimental data have been coupled to an analytical theoretical model, in order to quantify key parameters involved in the foci induction process. Induction of γ-H2AX foci was found to be affected by the initial radiation exposure with a smaller number of foci induced by subsequent exposures. This was compared to chromatin relaxation and cell survival. The time needed for full recovery of γ-H2AX foci induction was quantified (12 hours) and the 1:1 relationship between radiation induced DNA double strand breaks and foci numbers was critically assessed in the multiple irradiation scenarios.

## Introduction

Ionising radiation (IR) induces DNA damage both directly, through ionisation of the DNA backbone and indirectly, through the hydrolysis of water molecules producing free radicals, which can further react with, and damage, DNA [1-4]. Cells respond to IR, and the subsequent DNA damage, by activating a complex and well-organized set of biochemical signalling and effector pathways, known as the DNA damage response (DDR) pathway, which aims to restore the DNA to its original configuration, thereby maintaining genomic stability [1]. The most dangerous type of lesion is the DNA double strand break (DSB) i.e. a complete break of the DNA double helix. Extended DNA damage, such as staggered DSBs are difficult to repair and force cells to undergo apoptosis or cellular senescence resulting in clonogenic cell death [9]. This property of ionising radiation has been widely exploited in clinical practise to target and kill tumour cells through radiotherapy. As broken DNA ends are able to dissociate, DSBs are not only more difficult to repair, but also allow for the re-joining of unrelated ends, thus allowing for gross loss or amplification of genomic information, as well as chromosomal rearrangements, all of which are commonly associated with the early stages of cellular transformation and tumourigenesis [5]. Although it is now well accepted that many different processes are involved in the development of radiation induced cancer (such as epigenetic alterations and microenvironment modification [6-8]), it is still crucial to fully characterise the role of DNA damage and its repair following clinically relevant irradiation schedules in order to improve both cancer cell killing and healthy tissue recovery.

Although radiotherapy is an established practice currently used to treat nearly half of all cancer patients in the western world [10],basic radiobiological research continues to provide suggestions and evidences for further improvement and optimization [10]. One of the most common and powerful techniques used in modern radiotherapy is the fractionation of the total dose to which patients are exposed, into a set of exposures during which smaller doses are delivered separated by a recovery period of several hours (~12-24 hrs). Benefits of this dose splitting approach rely on both the tumour and healthy cell response to radiation. In terms of healthy or “normal” cells, dose fractionation allows repair of sub-lethal damage resulting in cellular survival and re-population of non-cancerous cells. In contrast, this technique forces re-oxygenation and re-assortment of tumor cells effectively enhancing the radiosensitivity of cancer cells to the subsequent radiation exposures [11]. 

Amongst the different markers of DNA DSBs, one of the most well characterized is the phosphorylation of the histone H2AX (γ-H2AX). Although it is commonly accepted that a γ-H2AX focus indicates the presence of a double strand break (DSB), while foci disappearance is associated with the repair of the DNA damage, the exact relationship between the number of foci and the number of DSBs is still a matter of debate [12-14]. Nonetheless, γ-H2AX is often used as a marker for exploring the spatial distribution and the DNA repair kinetics of cells following ionizing radiation exposure [14-18] and it also been suggested as a biomarker to predict patient response to specific radiotherapy treatments [19]. Following radiation exposure, histone H2AX is rapidly phosphorylated (within seconds) by the ATM and/or DNA-PK kinases at DNA DSB sites [20],, reaching a peak of H2AX phosphorylation at around thirty minutes after radiation exposure/DSB induction. This represents the maximum level of γ-H2AX foci detectable, which is directly linked to the absorbed dose and factors such as radiation quality, LET, cell type, dose rate, etc. [21]. Notwithstanding, in recent times it is becoming clear that H2AX phosphorylation might not only be important in terms of the sensing of DNA damage, but also in the chromatin remodelling process, which may play a critical role in DNA repair by allowing repair proteins to access the damaged regions of the DNA. Furthermore, recent studies have demonstrated the involvement of γ-H2AX in the cell death process, and in particular its crucial role in caspase independent apoptosis induction [22].

The main aim of this study was to investigate the temporal response and effectiveness of DNA repair processes when the radiation dose is delivered in a series of multiple acute exposures. AG01522 human fibroblasts were cultured *in vitro* and irradiated with 225 kVp and 30 kVp X-rays. The study focused on normal human fibroblasts to assess the response of typical H2AX phosphorylation response in healthy tissues where repair is of more interest and without the interference of cancer altered signalling pathways. The maximum number of γ-H2AX foci induced was found to be in the range 20–30 foci/Gy per cell nucleus for an acute single dose irradiation (dose rate ~0.6 Gy/min) which occurred at approximately 30 minutes post irradiation, which is in agreement with existing literature. After the thirty-minute peak, the overall number of foci decreases with an exponential trend following a kinetics model, as reported in previously [23]. Under these conditions, the induction and loss of γ-H2AX can provide information about how effectively the cellular DDR system reacts to acute external radiation-induced stimuli, providing parameters for how fast the H2AX histone is phosphorylated and dephosphorylated. As the number of ionizations and DSBs induced per unit dose by the subsequent radiation exposure is not expected to change [24], the γ-H2AX kinetics following the second radiation exposure can then provide information on the perturbation caused by the initial irradiation to the DNA repair machinery. These investigations have also been carried out with an altered primary dose (0.1 Gy) to test the perturbation of the γ-H2AX signalling pathway induced by a small dose. DNA damage induction and repair, as measured through the γ-H2AX assay, have then been related to changes in chromatin conformation (i.e. level of eu- and hetero-chromatin) and cellular survival in order to assess the biological relevance of the observed changes in the repair kinetics. Finally, the results obtained have been coupled with a phenomenological model that successfully describes and interprets the data, for both acute and split irradiations. The cellular DDR response to multiple exposures, which is of particular interest in radio-therapeutic context, shows an altered H2AX phosphorylation response, which is dependent on the time interval between irradiations. 

## Materials and Methods

### Cell Culture

Primary human AG01522 fibroblasts at low passage were acquired from Coriell Institute for Medical Research (Camden, NJ, USA). Cells were cultured in minimum essential media alpha MEM with deoxyribonucleosides (LONZA, UK) supplemented with 20% foetal bovine serum (FBS) and 1% penicillin/streptomycin. Cultures were maintained at 37 °C in an atmosphere of 95% humidified air and 5% CO_2_. Cells undergoing irradiation were transferred into six wells plate (10^5^ cells/well), containing glass coverslips and incubated overnight to allow cells to attach. Cells were 90% confluent at the time of irradiation and synchronized in G0/G1 phase of the cell cycle [25]. Where not otherwise specified, all cell culture reagents were purchased from Sigma-Aldrich Corporation (St Louis, MO, USA).

### Irradiation

Irradiations were performed using the X-ray cabinet of the CCRCB, at Queen’s University, in Belfast, UK (PXI X-Rays). Depending on the experiment type, the voltage was set to 225 kV_p_ or 30 kV_p_ - with a current of 45 mA (dose rate = 0.59 Gy/min at 50 cm from the X-ray tube for the 225 kV_p_ setup). The X-ray beam was hardened with a 2 mm removable copper filter which was not used for the 30 kV_p_ irradiations. Dosimetry was performed with a farmer chamber (PTW, Germany) cross calibrated with a secondary standard detector used in the Northern Ireland Cancer Centre. Dose uniformity within the exposed area (± 2%) was assessed using Gafchromic film RTQA^2^ Radiation exposures were conducted at room temperature with cells kept submerged in warmed culture medium (~ 2ml). Pre- and post-irradiation, cells were kept in a 37°C, 5% CO_2_ incubator in the same room. This minimized transfer time and possible stress caused by temperature and pH variations. Control experiments were performed following exactly the same irradiation protocol but without energizing the X-ray unit ("mock" irradiation, see Figure **S1**). 

### γ-H2AX detection

After irradiation, cells were fixed at different time points to study γ-H2AX induction and loss kinetics. Cells were fixed in ice cold 50% CH_3_OH and 50% (CH_3_)_2_CO for 20 minutes at room temperature. After fixation cells were permeabilized with 0.5% Triton X100:PBS and then blocked with 0.2% skimmed milk, 0.1% TritonX – 100, 5% FBS in Phosphate Saline Buffer (PBS). Cells were then stained with anti-γ-H2AX antibody (Upstate) and anti-mouse AlexaFluor-488 secondary antibody (Molecular Probes) for the kintecs experiments and with anti mouse AlexaFluor 568 (Molecular Probes) for the 53BP1/γ-H2AX colocalization experiments . Coverslips were mounted with *VECTASHIELD^®^* Mounting Medium containing DAPI, to counterstain cellular nuclei. γ-H2AX foci were scored manually by the same operator throughout the cell nuclei using a Zeiss Apotome fluorescence microscope with 63X objective and the average number of foci per cell was calculated from a minimum of 250 cells per dose/time point. Experimental data represent the average of 3 independent experiments. 

### 53BP1 detection

After irradiation, cells were fixed at different time points to study 53BP1 foci after radiation exposure. Cells were fixed in ice cold 50% CH3OH and 50% (CH 3)2CO for 20 minutes at room temperature. After fixation cells were permeabilized with 0.5% Triton X100:PBS and then blocked with 0.2% skimmed milk, 0.1% TritonX – 100, 5% FBS in Phosphate Saline Buffer (PBS). Cells were then stained with 53BP1 antibody (Novus Biologicals) and anti-mouse AlexaFluor-488 secondary antibody (Molecular Probes). Coverslips were mounted with VECTASHIELD® Mounting Medium containing DAPI, to counterstain cellular nuclei. 

### Hetero-/Eu-Chromatin detection

In order to evaluate the impact of the fractionated IR on the chromatin status (i.e. eu-chromatin vs hetero-chromatin), cells were grown on coverslips as previously reported and irradiated with indicated doses. Following irradiations cells were fixed at indicated time points using 4% paraformaldehyde:PBS. Samples were then stained with antibodies against acetylated histone H3 (acH3 Upstate) and heterochromatin protein 1 alpha (HP1α Cell Signalling Technology) to detect eu-chromatin and hetero-chromatin respectively. Coverslips were mounted using *VECTASHIELD^®^* Mounting Medium containing DAPI, to counterstain cellular nuclei. Cells were imaged using a Nikon Ti-S eclipse fluorescent microscope with a 63X objective. Quantification of the fraction of DNA in eu- or hetero-chromatin conformation was performed with Image J software using an intensity threshold algorithm set at 50% of the maximum signal from acH3 or HP1α. Fraction of chromatin status was calculated as relative to nuclear area as detected with DAPI staining. Data have been analyzed using a symmetric T-Student parametric test. 

### Cell survival

Cell survival was assessed using a conventional clonogenic assay. Cells were seeded in T75 flasks and exposed to known radiation doses at ~75-90% confluency. Immediately after the irradiation, cells were trypsinized, re-suspended in a single cell solution, counted and plated in multiwell plates at different densities. After 10 days incubation, the culture media was removed and cells stained with crystal violet (0.5%) in methanol for 30 minutes at room temperature. Survival fraction was calculated from the number of microscopic colonies scored using the 50 cell exclusion rule [21]. 

### Theoretical Model

The theoretical model, developed to analyze and quantify the mechanism underlying the dynamics of IRIF induction/decay, is based on an analytical approach that takes into account the foci phosphorylation and de-phosphorylation processes. The rational was to define a robust analytical function, able to reproduce the acute exposure behaviour and hence using this class of functions to analyze the split dose scenario. The function adopted in this work is the product of two terms representing the competitive process of foci induction and decay after an acute irradiation. The induction process has been modelled with function saturating at the maximum level of foci induction, as shown in eq. (1)

$$N(t)=A(1-e^{-Bt})$$ (1)

where A represents a normalization factor and B drives the dynamics of the IRIF induction. Simultaneous to this induction process, the experimental evidence suggests that IRIFs disappear accordingly to a decay trend. In accordance with previously reported data [26,27], we hypothesized a two phase decay process, with two different constant rates, i.e. slow repair kinetics (which is thought to be linked to repair of complex DNA damage) and fast repair kinetics ( linked to simple DNA damage). The function adopted for the decay is therefore:

$$N(t)=(Ce^{-Dt}+(1-C)e{-}^{-Et})$$ (2)

where C represents the amount (weight) of simpler damage (with its correspondent decay rate D), whereas (1-C) represents the more complex damage with a decay rate E. 

**Figure 1:**
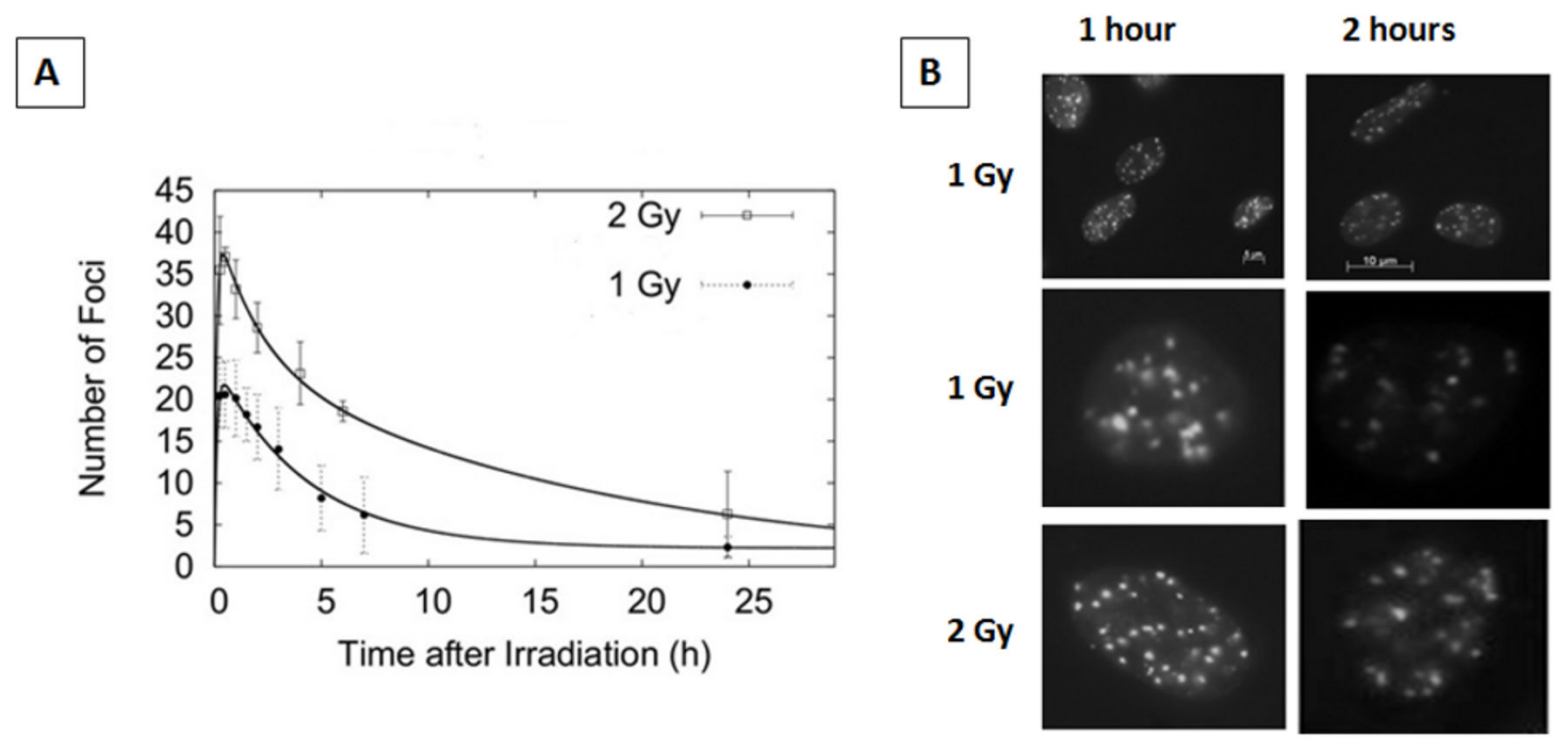
Foci kinetics following an acute irradiation with 225 kV_p_ X-rays. Panel A. Experimental data and fit (from eq. 3) of γ-H2AX foci after exposure to 1 Gy (full circles) and 2 Gy (open squares) for 225 kV_p_ X-rays. Error bars represent one standard error of the mean of 3 independent experiments. Panel B. Representative pictures of γ-H2AX foci after irradiation with 1 and 2 Gy of 225 kV_p_ X-rays. Upper figures represent 2 examples of a small field of view, whereas the middle and lower pictures show view at a single cell level.

The resulting final equation describing the N of foci at a specific time post acute radiation exposure is therefore:

$$N(t)=A(1-e^{-Bt})(Ce^{-Dt}+(1-C)e^{-Et})$$ (3)

and it has been used to fit the experimental data in Figure 1.

This set of functions has also been used to describe the IRIF behaviour in the split dose scenario. The function adopted in the split dose scenario is the independent sum of two acute exposure functions (same class of equation 3) with the first one starting at time t = 0 and with fixed parameters obtained from fitting single acute exposures , and the second equation with free parameters starting at the time of the second irradiation. The mathematical formalization is the following

$$N(t)=\left\{\begin{array}{c} \alpha (1-e^{-\beta t})(\gamma e{-}^{\delta t}+(1-\gamma )e^{-\varepsilon t})\\ \alpha (1-e^{-\beta t})(\gamma e{-}^{\delta t}+(1-\gamma )e^{-\varepsilon t})+A(1-e^{-B(t-\Delta t)})(Ce^{-D(t-\Delta t)}+(1-C)e^{-E(t-\Delta t)}) \end{array}\right\}$$ (4)

where α, β, γ, δ, ε are the (fixed) parameters as obtained from the single acute exposure scenario and A, B, C, D, E are the parameters related to the second irradiation only.

## Results

### γ-H2AX response after acute dose exposure

**Figure 2:**
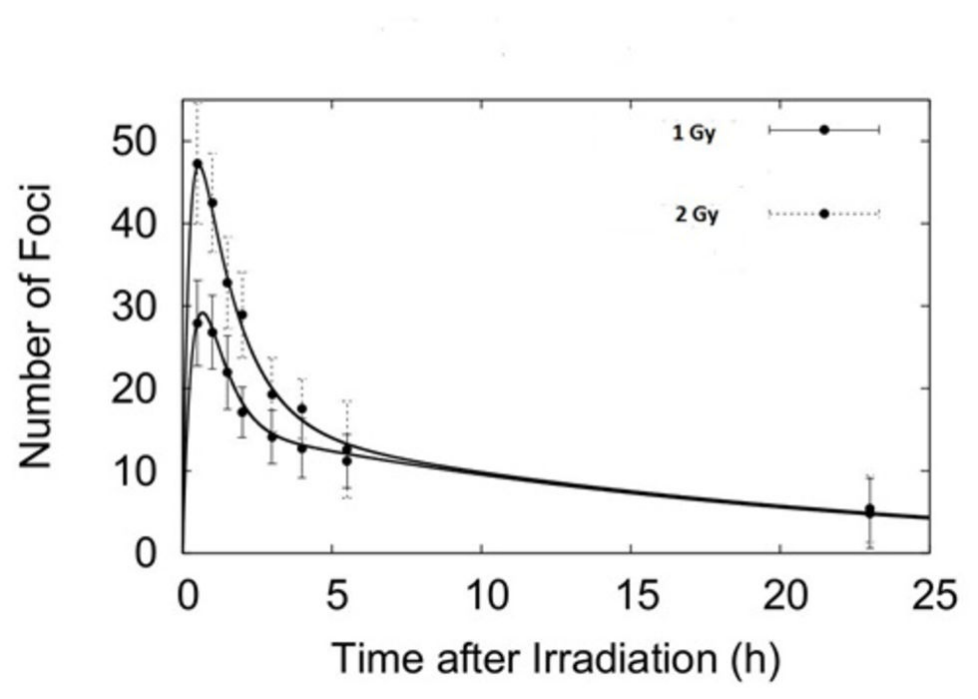
Foci kinetics following an acute irradiation with 30 kVp X-rays. Experimental data and fit (from eq. 1) of γ-H2AX foci after exposure to 1 Gy (full circles) and 2 Gy (dotted circles) for 30 kV_p_ X-rays. Error bars represent one standard error of the mean of 3 independent experiments.

Initially, we have characterized the γ-H2AX foci induction kinetics in our cell line model following a single acute radiation exposure. Cells were irradiated with X-rays of two different peak energies; 30 and 225 kV_p_ with 1 and 2 Gy at a dose rate of ~0.6 Gy/min. In particular, we were interested in evaluating the effect of radiation dose, quality and possible saturation on the detection of foci within each cell nucleus. Using these two different peak energies, we found that the maximum number of γ-H2AX foci is reached at 30 minutes after irradiation (with ~21 and 37 foci/cell for the 1 Gy and 2 Gy exposures respectively) after which it continuously decreases up to 24 hours (Figures **1** & **2**). Considering the experimental uncertainties, it is possible to conclude that the saturation effect (i.e. reaching a maximum level of detectable foci due to physical/imaging foci overlapping) plays a negligible role in the dose range investigated (0-2 Gy). Additionally, the use of the Apotome microscope allowed us to accurately score foci in 3D, further reducing the probability of underestimating foci counts due to physical overlap through the z-axis. The number of foci per cell per Gy is higher following the 30 kVp X-ray irradiation than the 225 kVp in line with the expected higher biological effectiveness of lower energy X-rays [28]. In both cases, the foci appeared randomly distributed across the cell nucleus and of similar size to endogenous, background foci in untreated cells (Figure **1**). The acute exposure foci dynamics have been fitted using equation 3 obtaining good agreement at both 1 and 2 Gy.

### γ-H2AX response after multiple split dose exposures

**Figure 3:**
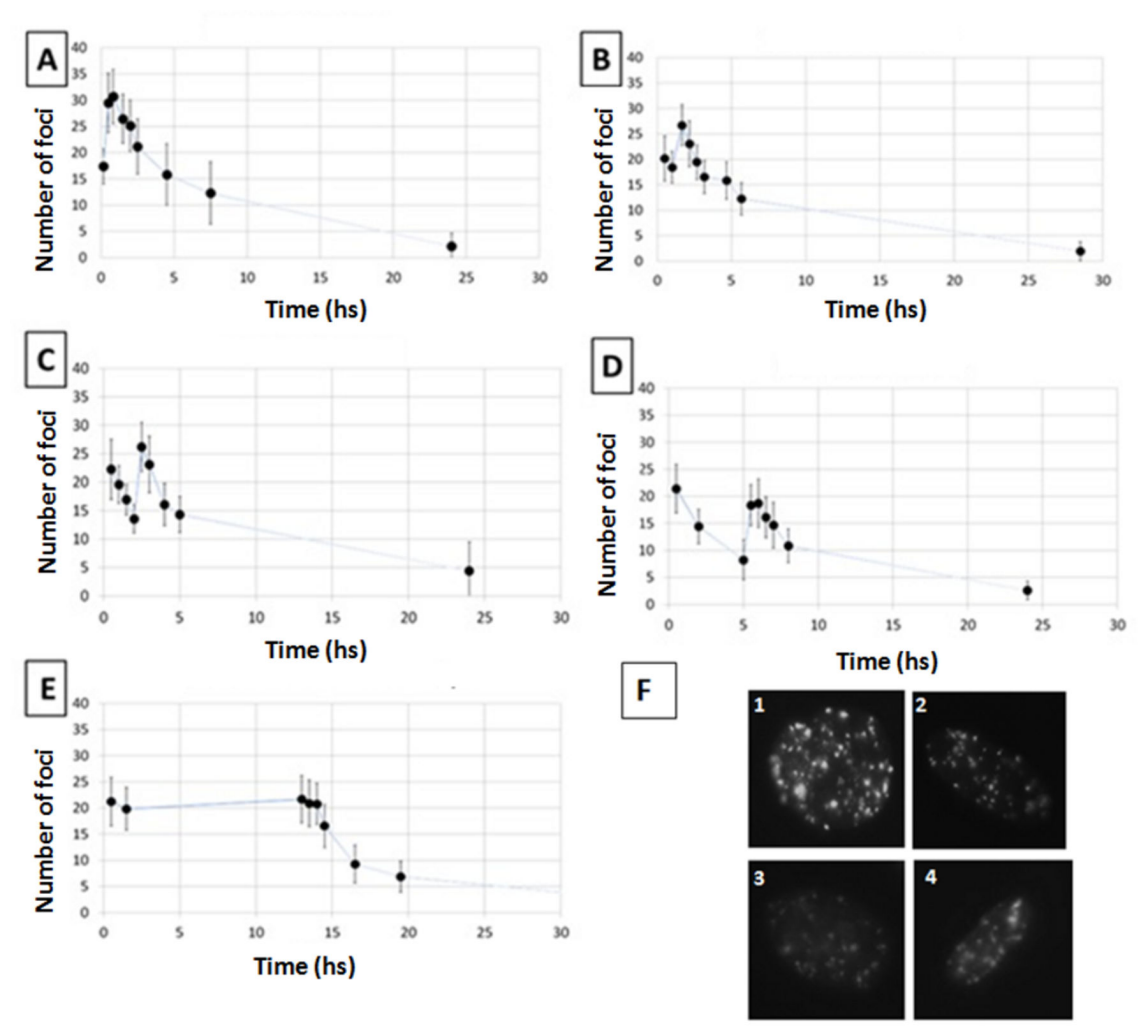
γ-H2AX foci kinetics following split multiple radiation exposures. Number of γ-H2AX foci per cell after exposure to 1 Gy at time 0 plus 1 Gy delivered20 min later (panel A), 1 hour later (panel B), 2 hours later (panel C), 5 hours later (panel D) and 12 hours later (panel E) using 225 kV_p_ X-rays. In Panel F representative pictures of cells in different exposures scenario are presented, i.e. cells fixed after 30 minutes in the cases of 20 minutes split dose exposure (Figure 1), 1 hour split dose exposure (Figure 2), 2 hours split dose exposure (Figure 3) and 5 hours split dose exposure (Figure 4). Error bars represent one standard error of the mean of 3 independent experiments. Lines are guides for the eyes.

**Figure 4:**
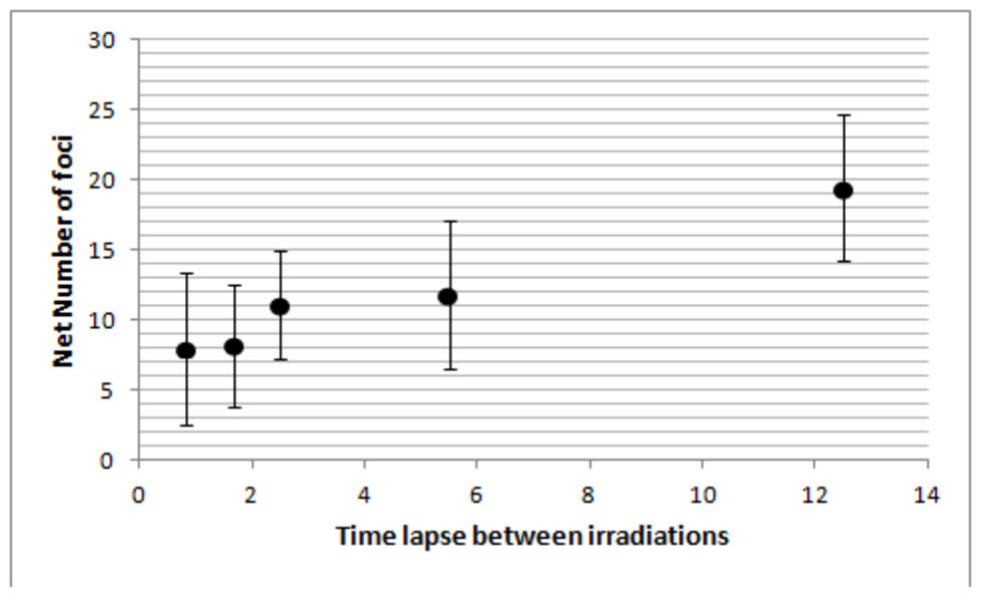
Net number of γ-H2AX foci induced by the second radiation exposure. Estimated number of γ-H2AX foci induced by the second irradiation only as calculated by subtracting the predicted residual number of foci from the first exposure (using the single irradiation kinetic data) from the total number of foci measured 30 minutes after the second irradiation.

After establishing the γ-H2AX foci dynamics for the acute irradiation, we investigated the split dose scenario. Cells were irradiated with 2 Gy of 225 kV_p_ X-rays split into 2 exposures of 1 Gy each with varied time intervals between the two exposures, from 20 minutes up to 12 hours (Figure **3**). Cell were then fixed and stained at different times (30 min to 24 hrs) after the second radiation exposure. Data show a single peak of ~30 foci/cell for split irradiations with a 20 min gap whilst two separate peaks are evident when the recovery time between exposures is 1 hour or longer. In order to evaluate the effectiveness of the second irradiation in the induction of γ-H2AX foci, we have subtracted the residual foci from the total number of foci scored 30 minutes after the second irradiation. This was achieved using equation 3 to estimate the number of residual foci from the 1^st^ exposure at the time of the second irradiation (Figure **4**). With less than 5 hours gap between irradiations, data indicate a small increase in the number of foci caused by the second exposure whereas after 12 hours the second irradiation induces a number of foci comparable to that obtained following a single acute irradiation. 

**Figure 5:**
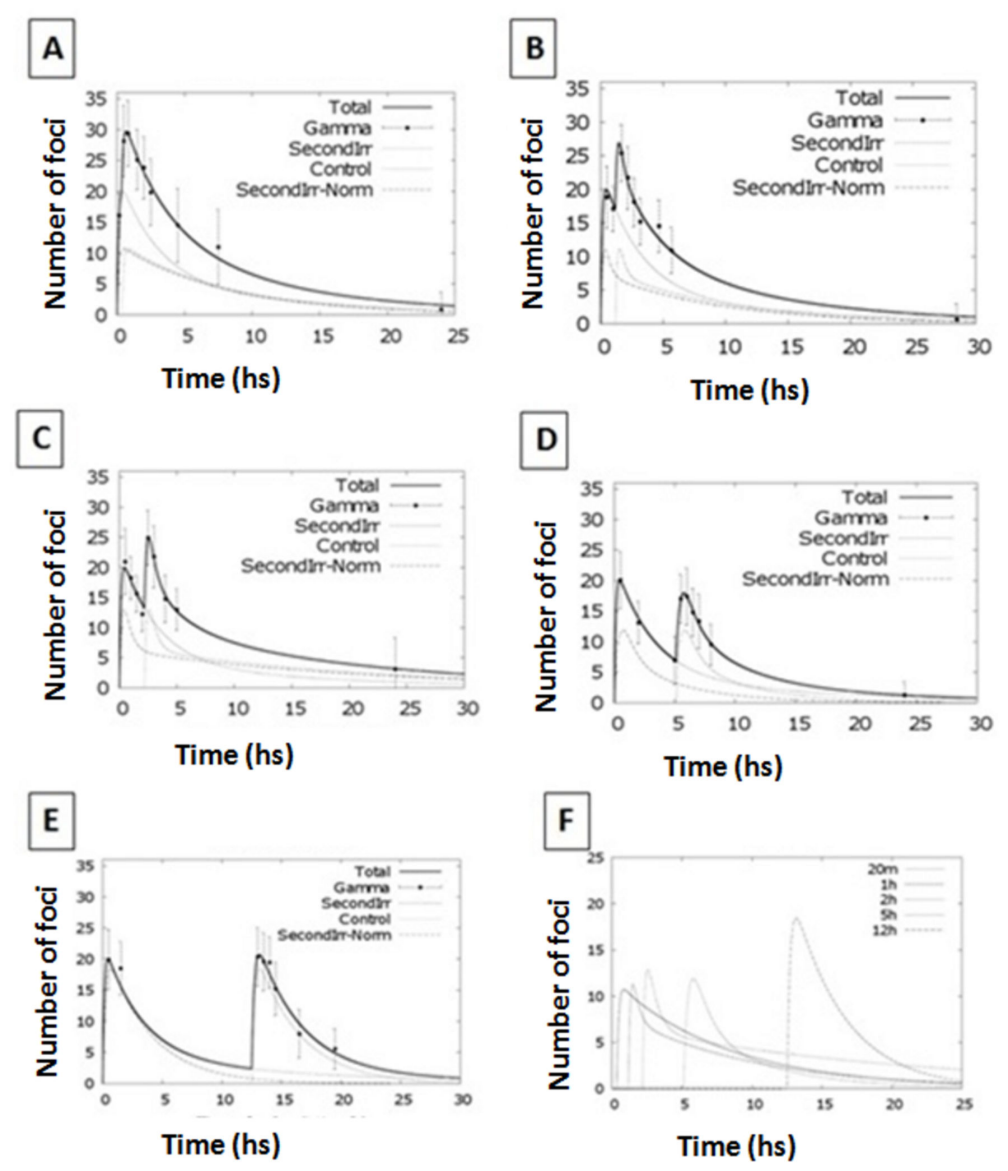
Fitting kinetics for split dose irradiation exposures. Number of γ-H2AX foci per cell after exposure to 1 Gy at time 0 plus 1 Gy delivered 20 minutes, 1 hr, 2 hr, 5 hr or 12 hr later using 225 kV_p_ X-rays. Solid line represents the modelling function for the split dose scenario. Dotted lines represent the modelling function for the individual radiation exposures. Kinetics for the second exposure only are reported in the last panel.

Equation 4 was then also used to fit the entire foci kinetic range, in order to gain information on the behaviour of the cellular DDR system after split irradiations. Having defined the parameters for the initial exposure (from the single acute irradiation experiments), we treated the second irradiation as a perturbation occurring after the specified time interval (i.e. time gap between exposures). Data fitting provided parameters for quantification of the IRIF dynamics following the second irradiation. The modelling analysis revealed two different foci kinetics when the radiation exposures are within 5 hrs. (Figure **5** and Table **S1**). After a 12 hours interval, the system appears no longer perturbed and the IRIF induction/repair kinetics of the second irradiation display similar characteristics to those of a single dose acute exposure. 

In order to test the robustness of our experimental model in this fractionated scenario we investigate the response of other marker of DSBs, i.e. 53BP1 protein recruitment at the damage site. 53BP1 is a pivotal player of the processing and repair of double-strand breaks, acting downstream of γ-H2AX-dependent hierarchy of proteins [29-32]. In particular we investigated the induction of 53BP1 foci induced by radiation in the same exposure scenario adopted for the gamma H2AX scoring. From the results showed in Figure **S2** and **S3**, the 53BP1 foci co-localize with the γ-H2AX. Images show (qualitatively) a notable decrease in the number of 53BP1foci in agreement with the γ-H2AX response following acute and split dose experiments (1 and 6 hours gap)

### γ-H2AX and cell survival response after multiple split dose exposures

**Figure 6:**
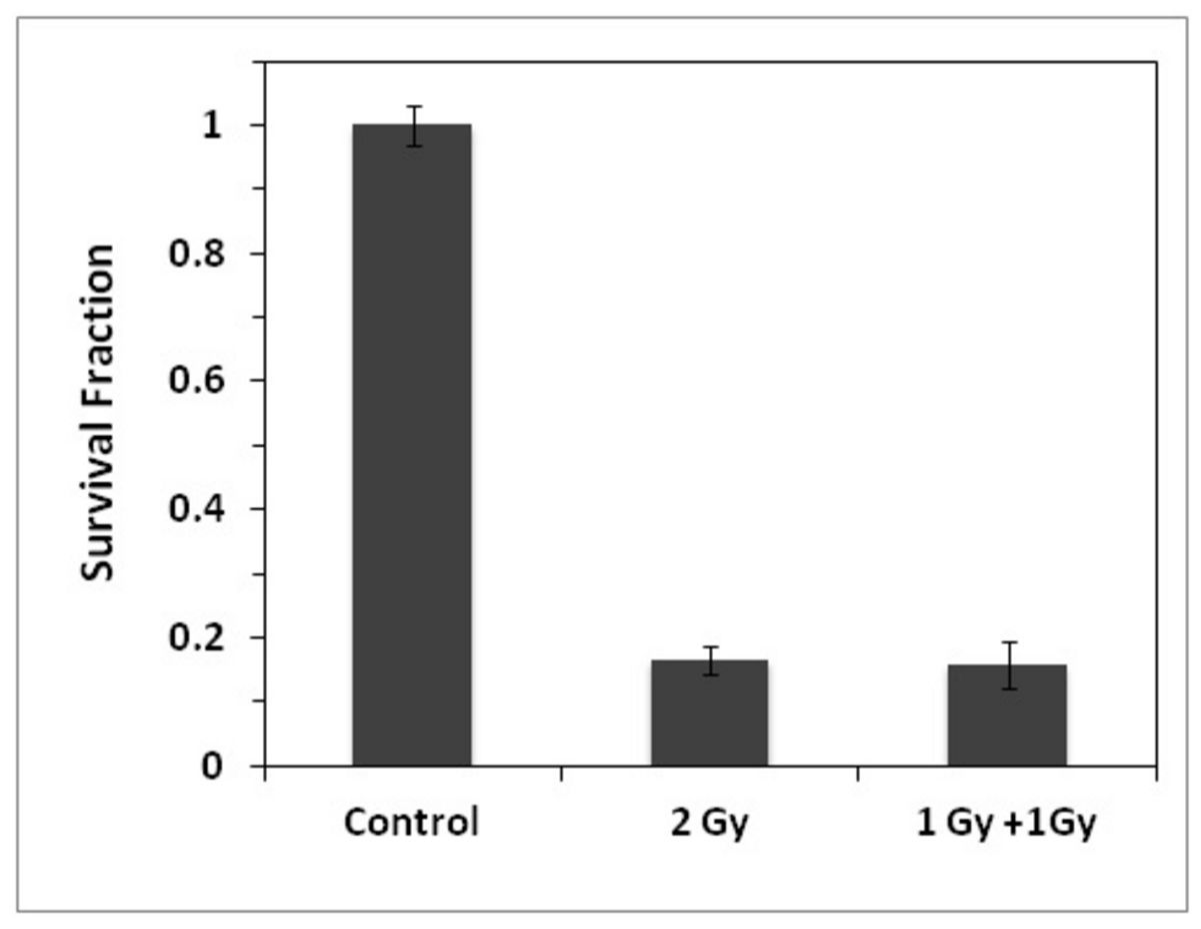
Clonogenic data survival following single and split dose irradiations. Comparison of clonogenic survival following acute (2 Gy) and split dose (1 Gy + 1 Gy) exposures with 1 hr time gap. Error bars represent the standard error of the mean of 3 independent experiments.

Interestingly, the experimental data (supported by the statistical analysis) indicates a reduced number of foci induced by the second exposure if this occurs within 5 hrs from the first one. According to the hypothesis of a 1:1 relationship between foci and DSBs, this should then result in a reduced cell killing effect for the split irradiations (within 5 hours). Although a reduced cell killing effect is well documented for recovery periods of several hours (due to repair of sub-lethal lesions) and indeed constitutes the fundamental basis for the fractionation technique in radiotherapy, it should not have a significant cellular impact when radiation exposures are so closely spaced (< 5 hrs). To verify this, we performed standard clonogenic assays to assess the cell killing effect induced by a split irradiation with a time gap of 1 hr (**Figure 6**). As expected, the cell killing effect of a 2 Gy acute exposure is similar to that obtained by splitting the dose into two equal fractions of 1 Gy with only 1 hr recovery gap, suggesting that the number of foci detected after a split dose irradiation may not be reflective of the effective level of damage induced. Although clonogenic and DNA damage data do not appear agree (i.e. 2 Gy acute exposure induces ~37 foci/cell compared to the ~27 foci/cell of the 1 Gy + 1 Gy split irradiation with both the acute and split dose exposures causing the same level of cell killing), it must be noted that the repair kinetics of the second exposure also appear slower (Figure 3) if the two irradiations are close in time.

### Eu/Hetero chromatin response after multiple split dose exposures

**Figure 7:**
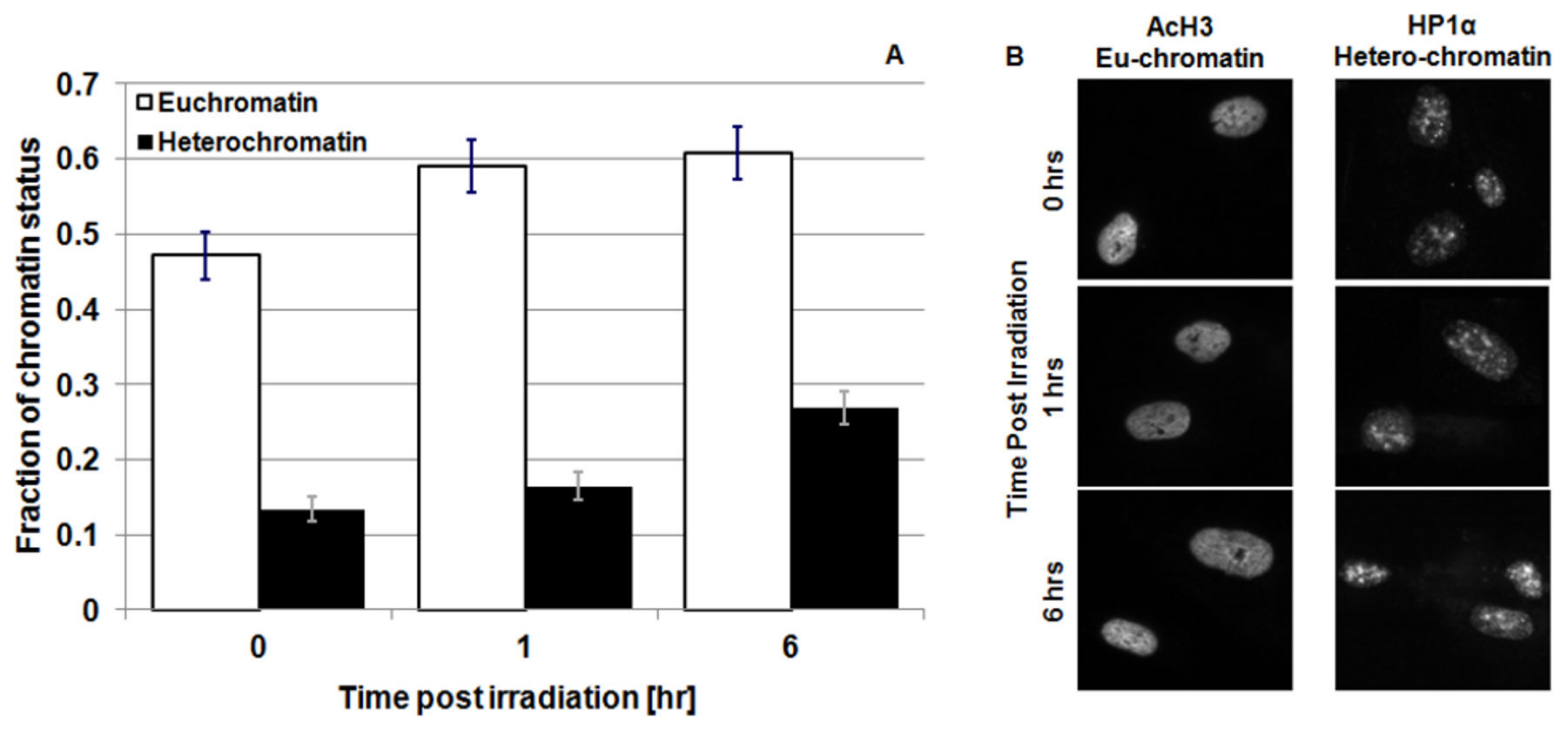
Eu/hetero-chromatin change following radiation exposure. Panel A: Amount of eu- (acH3 staining - light bars) and hetero-chromatin (HP1α. staining - dark bars) in cell nuclei as a function of time after 1 Gy of 225 kV_p_ X-rays irradiation of 3 independent experiments. The analysis of the experimental data has been performed with a symmetric T-Student test. Panel B: Representative picture of eu- and hetero-chromatin staining .

Recent investigations have highlighted the key role played by chromatin status in the repair of DSBs [33,34]. In particular, there is some controversy regarding the phosphorylation of H2AX in highly condensed DNA regions (i.e. heterochromatin) [35,36] and the chromatin dynamics associated with repair of DNA DSBs [37-40]. It is however, accepted that following the induction of DNA lesions, chromatin undergoes considerable changes in conformation in order to trigger and favour specific DNA repair mechanisms and allow repair proteins to access the DNA lesion. As these changes can occur relatively rapidly (e.g. within minutes following radiation exposure), this may also impact on the induction and/or detection of DSBs caused by a secondary irradiation, by affecting either the phosphorylation of H2AX and/or the staining of the phosphorylated histones. In order to test whether changes in chromatin structure may be linked to the different number of foci observed following split exposures, we monitored the fraction of hetero- and eu-chromatin at different times post irradiation. This was carried out by staining irradiated cells with antibodies against acetylated histone H3 (AcH3), and heterochromatin protein 1α (HP1α), which are markers of euchromatin and heterchromatin respectively (Figure **7**). We observed a significant increase in eu-chromatin at 1 hr post irradiation. This is consistent with the accepted model of global relaxation of DNA after irradiation which is thought to help facilitate DSB repair [41]. The increase in the amount of eu-chromatin is persistent at 6 hr post irradiation, while no statistically significant change in hetero-chromatin is observed. However, it has to be noted that the probes used for assessing chromatin status (i.e. acH3 and Hp1α) are only specific for defined eu- and hetero-chromatin domains. For the hetero-chromatin in particular, there may be regions which are not being accounted for in the present analysis. As the vast majority of hetero-chromatin is marked by Hp1α, the changes observed are adequate to test the specific hypothesis. 

### γ-H2AX response after adaptive response exposures

**Figure 8:**
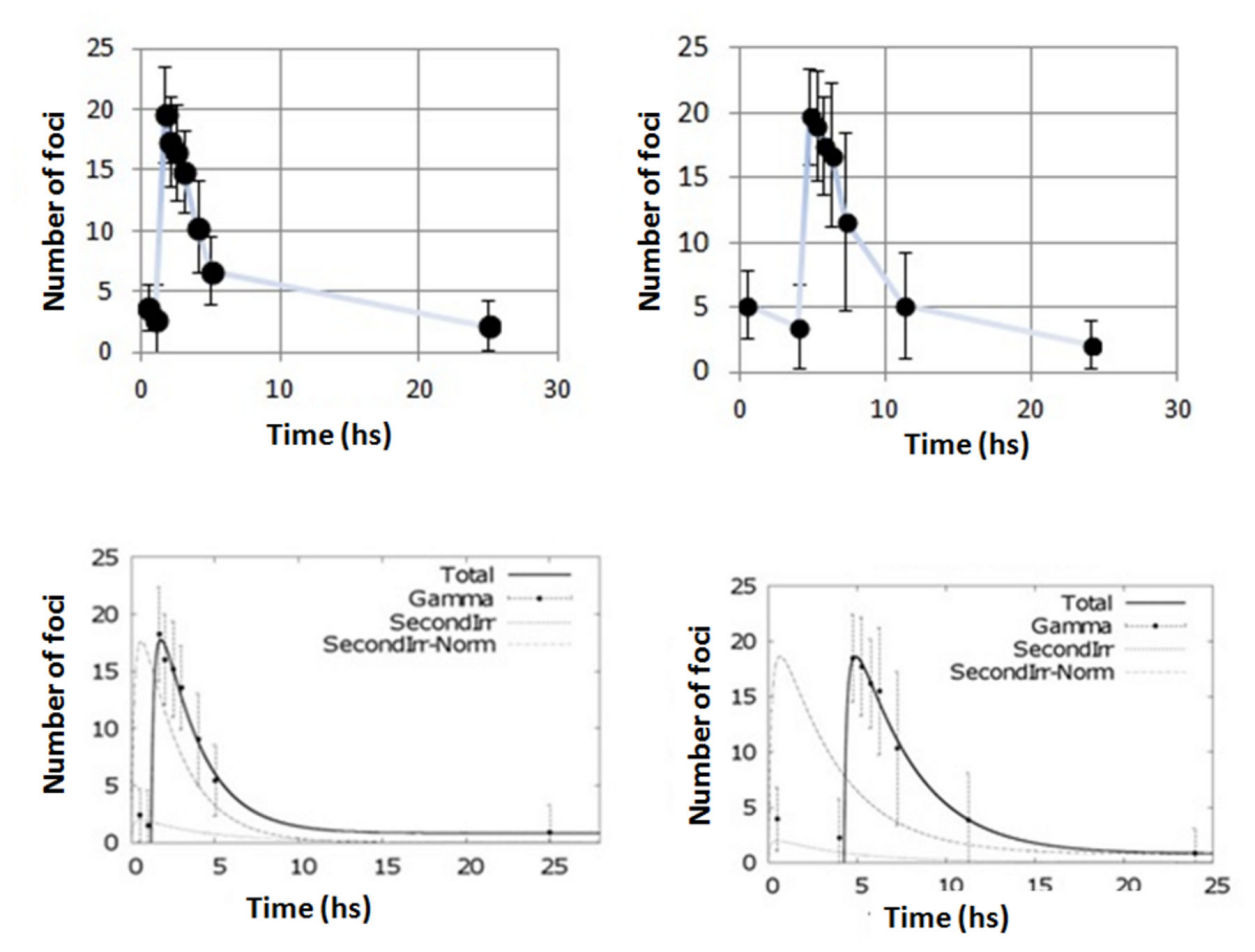
Split dose irradiations with small initial dose. Number of γ-H2AX foci after exposure to 0.1 Gy at time 0 plus 1 Gy delivered 1 hr after (left column) and 4 hr later (right column). Top panels report the experimental data;bottom panels, data are fitted using the modelling equation (4). In the bottom panels, the solid line represents the modelling function for the split dose scenario. The dotted lines represent the modelling function for the individual radiation exposures. Error bars represent the standard error of the mean of 3 independent experiments.

Having assessed the perturbation of the γ-H2AX response following a 1 Gy initial irradiation, we focused our attention on the level of X-rays dose required to perturb the γ-H2AX response system and trigger a smaller foci response of following irradiations. Cells were therefore irradiated with an initial dose of 0.1 Gy (conventionally accepted as threshold for low dose effects [42]) followed by 1 Gy and the induction and disappearance γ-H2AX foci was monitored (Figure **8**). Data collected revealed that a small initial dose (0.1 Gy) does not seem to significantly perturb the system, even if the irradiations are very close in time (1 hour). 

## Discussion and Conclusions

The aim of this work was to characterize the response of the H2AX phosphorylation system as a possible marker for DNA damage response and repair kinetics following single and split-dose irradiations. In particular, we focused our attention on the impact of the recovery gap between irradiations and the development of a mathematical model able to describe both acute and split dose exposures and assess changes in the cellular repair processes. The work was performed in normal human fibroblasts with a typical ATM and H2AX phosphorylation response [43]. From a clinical prospective, repair is more critical in healthy tissues as a large amount of lethal DNA lesions is expected to be induced in cancer cells, which often have perturbed DDR systems, whilst gaps between irradiations provide an opportunity for normal cells (with intact DDR systems) to repair the insult. The analysis of H2AX phosphorylation kinetics in response to a single acute dose of IR, indicates a peak of H2AX phosphorylation at 30 minutes post exposure with an average number of ~25 foci per cell nucleus per Gy using 225 kV_p_ X-rays. The resulting foci kinetics follow a clear negative exponential response and very little residual damage was detected after 24 hr post exposure. These results are in good qualitative agreement with those found in the literature [9]. 

In contrast split dose experiments show that the γ-H2AX response following the second radiation exposure is time-lapse dependent, with the number of foci induced inversely proportional to the time interval between exposures. This effect disappears with irradiation gaps >5 hrs, after which the two irradiation responses behave as independent acute insults. The lower number of foci induced by the second exposure cannot be attributed to a saturation issue (either related to foci overlap and microscopy limitations or to the phosphorylation signal) as considerably larger numbers of foci are detected in these cells following higher doses (i.e. 2 Gy single exposure). Additionally, the good linearity reported between the number of foci induced by 1 Gy and 2 Gy acute exposures is also an indication of the suitability of this methodology for assessing DNA damage following radiation exposure and is supportive of the use of the γ-H2AX assay as a radiation dosimeter, at least for acute exposures. 

It is thought that heterochromatin formation may protect DNA from radiation damage (by excluding water molecules from DNA, thereby reducing the amount of DNA damage caused by radiation induced radical species), and/or prevent H2AX phosphorylation by reduces physical access to histones. Therefore, to investigate whether increased heterochromatin may account for the reduced γ-H2AX response following the initial radiation we examined heterochromatin/euchromatin status in our cell line model following the initial radiation exposure. However, we did not observe any significant increase in the fraction of heterochromatin following radiation. On the contrary, our data indicate a relaxation of the DNA structures following radiation-induced damage, which is consistent with published models [32]. Moreover, clonogenic survival data confirm that the same level of DNA damage (and same cellular response) is induced by an acute exposure as is when the same dose is split into two temporally close irradiations (<5hours gap between exposures). Therefore, as the level of damage (i.e. DSBs induced) per unit dose must be the same between the two exposures, the difference in number of foci detected must be related to the detection and/or efficiency of the γ-H2AX phosphorylation system. In fact, when irradiations are closely spaced in time, the γ-H2AX systems does not represent a good marker for DSBs. 

Fitting of the experimental data with the proposed model (equation 4), indicates that γ-H2AX foci formation always follows similar kinetics (i.e, similar rise of the curves as observed in last plot of Figure 5, with β values in Table S1 very similar for all the irradiation settings). However, the induction of foci following the second irradiation appears to be slower than that caused by the first exposure (i.e. B parameter smaller than β). Additionally, the foci disappearance kinetics suggests that DNA repair is significantly slower following the second irradiation if this occurs within 5 hrs after the first irradiation. These data suggest a reduced efficiency of the DDR system when cells are already in the perturbed state induced by a single acute dose of 1 Gy. In other words, it is possible that when the system (cell) is unperturbed (t = 0), the DNA damage response system is at its most sensitive and the phosphorylation of the H2AX plays a central role in DNA damage signalling, with a 1:1 foci-DSB relationship as widely reported in the literature. In contrast, once the system has been perturbed (i.e. substantial DNA lesions induced by an initial radiation exposure) and the DDR machinery has been activated, phosphorylation of the H2AX histone may not be critically relevant for activation of the DDR system. Additionally, DSBs induced in the background of an activated DNA damage response might not be processed through the same mechanisms/pathways (as supported by the slower repair kinetics of second irradiations). This is consistent with reports that H2AX phosphorylation acts as a DNA damage signal amplifier and is crucial for damage recognition and/or efficient DNA repair [33]. According to such hypothesis, the initial radiation exposure may activate DNA damage signal amplification through H2AX phosphorylation to a level sufficient to trigger robust DDR activation and initiate DNA repair. Once the DNA repair machinery is activated, further phosphorylation of H2AX is no longer a critical requirement for DDR activation, resulting in reduced foci formation by subsequent radiation insults. If true, phosphorylation of the H2AX histone may not be a required/key step within the DDR in cells in which the DDR has been “pre-activated” or “primed” thereby invalidating the 1:1 foci-DSB correlation. Subsequent DSBs which do not result in a γ-H2AX foci are still processed by the cellular DSB repair machinery (alternatively we would observe a significant increase in cell killing for the split dose exposures). This has been previously reported in H2AX -/- cells in which DSBs are still repaired, albeit less efficiently, by the canonical DSB repair machinery. The system seems to reset after approximately 6 hrs from the initial perturbation event. These results, combined with our results from split dose experiments carried out with small initial doses (0.1 Gy), support our hypothesis that a threshold level of H2AX phosphorylation is required for efficient DDR activation/DNA repair. In contrast, following small dose exposures (<0.1 Gy), the DNA damage induced is very low and does not cause a significant perturbation of the system i.e. H2AX phosphorylation does not reach its ‘threshold’ level with the overall level of phosphorylated H2AX still low at the time of the second exposure. The subsequent 1 Gy irradiation, therefore, is perceived as an isolated insult triggering the same response as a single acute exposure. As described, H2AX phosphorylation following ionizing radiation is carried out primarily by the ATM kinase and also redundantly by the DNA-PK kinase. Additionally, activation of these kinases requires DNA end binding by the Mre11:Rad50:Nbs1 (MRN), and Ku70:Ku80 (Ku) complexes respectively [2]. Therefore, it is likely possible that following a secondary radiation exposure, activation of these kinases may be reduced and/or levels of, and/or the DNA binding capacity of, the MRN or Ku complexes may be reduced following the primary radiation resulting in reduced H2AX phosphorylation. Additionally, a plethora of additional proteins exist that regulate the activities of ATM and/or DNA-PK following IR e.g. the PP2A and PP6 phosphatases, the activities of which may also be affected in a split dose scenario resulting in altered H2AX phosphorylation/repair kinetics[44,45]. Thus, it is clear that further studies will be required in order to understand the exact mechanism(s) underpinning the reduction in H2AX phosphorylation following a secondary irradiation. Nevertheless, H2AX phosphorylation is a key focal node within the ATM and DNA-PK pathways and hence, is being increasingly used as a DNA damage/repair biomarker following radiation exposure . 

On the basis of the data presented and in the context of the cell model employed, the γ-H2AX assay appears to be a good and reliable indicator of the number of DSBs being induced, and repaired, following a single acute radiation expsoure. However, once the biological system is perturbed and the DNA damage response/DNA repair mechanisms are activated, phosphorylation of H2AX appears to change, both in terms of number of foci formed per unit dose absorbed, and in the rate at which foci are resolved. The response is restored after 6 hrs from the initial radiation insult. These data suggest that H2AX foci induction/disappearance might not be a universally reliable indication of DNA damage and/or repair. Additionally, although our data clearly indicate a non-cumulative response of the γ-H2AX phosphorylation and stress the impact of split dose exposures on the DNA repair machinery, the clinical relevance of these findings will have to be validated for the specific cell lines and 3D/*in-vivo* models in which the γ-H2AX assay is proposed as a biomarker.

## Supporting Information

Figure S1 **Background level of γ-H2AX after mock irradiation.** Number of γ-H2AX foci in AG01522 cells exposed to mock irradiation. Time 0 represents the moment of the (mock) irradiation. The data are obtained after 3 independent experiments and the error bars represent the standard error of the mean.
(TIF)

Figure S2 **Co-localization of γ-H2AX and 53BP1 foci after single and split irradiations (fixed after 30 minutes after irradiation).** γ-H2AX and 53BP1 pictures taken after 30 minutes of irradiation for: (first row) Single Dose exposure, (second row) Split dose with a time interval of 1 hour. The images are taken 30 minutes after the 2^nd^ exposure. (third row) Split dose with a time interval of 6 hour. The images are taken 30 minutes after the 2^nd^ exposure.
(TIF)

Figure S3 **Co-localization of γ-H2AX and 53BP1 foci after single and split irradiations (fixed after 1 hour after irradiation).** γ-H2AX and 53BP1 pictures taken after 30 minutes of irradiation for: (first row) Single Dose exposure, (second row) Split dose with a time interval of 1 hour. The images are taken 30 minutes after the 2^nd^ exposure. (third row) Split dose with a time interval of 6 hour. The images are taken 30 minutes after the 2^nd^ exposure.
(TIF)

Table S1 **Second exposure fitting parameters.** Parameters describing the repair kinetics of the different radiation exposure modalities as obtained by Equation (4).
(DOCX)
