# Supplementary figures and images for: Use of the γ-H2AX Assay to Investigate DNA Repair Dynamics Following Multiple Radiation Exposures
Source: PLoS One. 2013 Nov 29;8(11):e79541. doi: 10.1371/journal.pone.0079541 (PMC3843657; doi:10.1371/journal.pone.0079541)

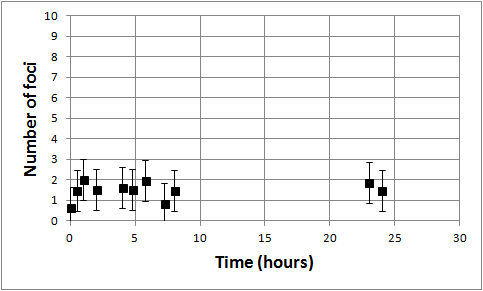

Supplement: Figure S1 — Background level of γ-H2AX after mock irradiation. Number of γ-H2AX foci in AG01522 cells exposed to mock irradiation. Time 0 represents the moment of the (mock) irradiation. The data are obtained after 3 independent experiments and the error bars represent the standard error of the mean. (TIF) [file pone.0079541.s001.tif]

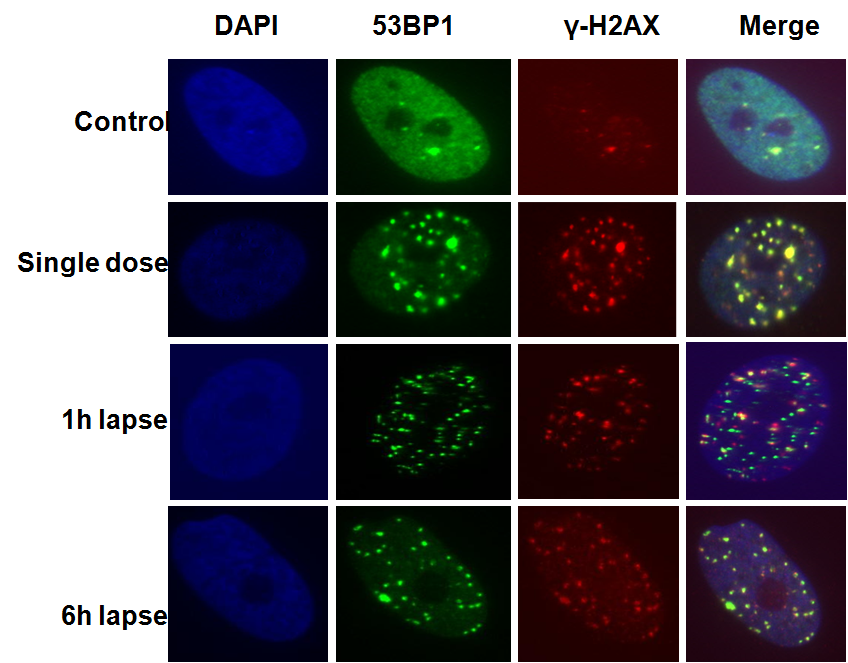

Supplement: Figure S2 — Co-localization of γ-H2AX and 53BP1 foci after single and split irradiations (fixed after 30 minutes after irradiation). γ-H2AX and 53BP1 pictures taken after 30 minutes of irradiation for: (first row) Single Dose exposure, (second row) Split dose with a time interval of 1 hour. The images are taken 30 minutes after the 2nd exposure. (third row) Split dose with a time interval of 6 hour. The images are taken 30 minutes after the 2nd exposure. (TIF) [file pone.0079541.s002.tif]

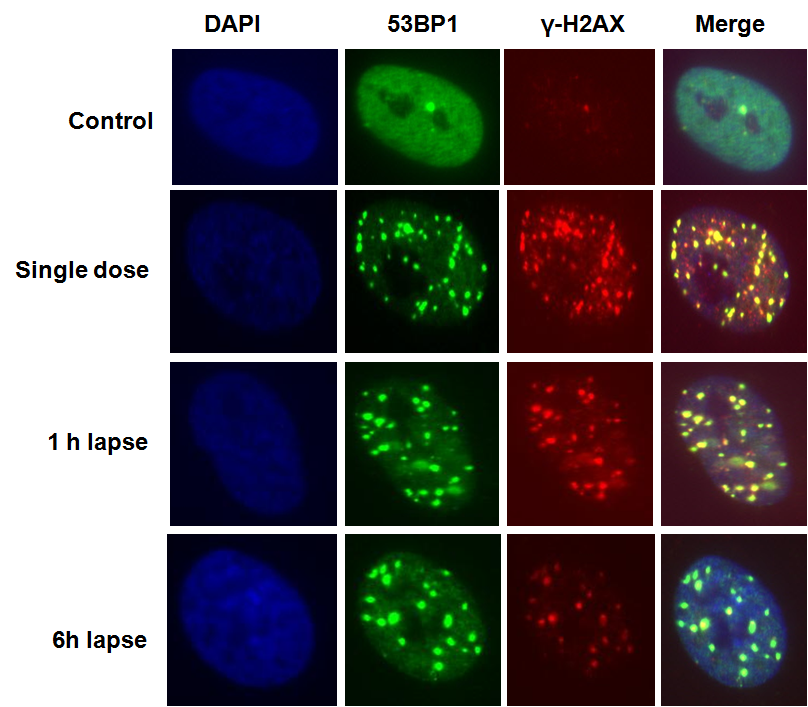

Supplement: Figure S3 — Co-localization of γ-H2AX and 53BP1 foci after single and split irradiations (fixed after 1 hour after irradiation). γ-H2AX and 53BP1 pictures taken after 30 minutes of irradiation for: (first row) Single Dose exposure, (second row) Split dose with a time interval of 1 hour. The images are taken 30 minutes after the 2nd exposure. (third row) Split dose with a time interval of 6 hour. The images are taken 30 minutes after the 2nd exposure. (TIF) [file pone.0079541.s003.tif]
