# Supplementary material for: Use of the γ-H2AX Assay to Investigate DNA Repair Dynamics Following Multiple Radiation Exposures
Source: PLoS One. 2013 Nov 29;8(11):e79541. doi: 10.1371/journal.pone.0079541 (PMC3843657; doi:10.1371/journal.pone.0079541)
Supplement: Table S1 — Second exposure fitting parameters. Parameters describing the repair kinetics of the different radiation exposure modalities as obtained by Equation (4). (DOCX) [file pone.0079541.s004.docx]

**Supplementary material**

Table S1

| Single Acute Dose | A | B[t] | C | D [t^-1^] | E [t^-1^] |
| --- | --- | --- | --- | --- | --- |
| 1 Gy | 24.63 | 8.011 | 0.91 | 0.23 | 3.32E-12 |
| 2 Gy | 41.67 | 9.55 | 0.41 | 0.50 | 0.06 |
|  |  |  |  |  |  |
| Radiation Gap | α | β [t] | γ | δ [t^-1^] | ε [t^-1^] |
| 20 Min | 100.9 | 0.69 | 0.15 | 2.55 | 0.15 |
| 1 hour | 27.7 | 3.93 | 0.73 | 2.74 | 0.11 |
| 2 hours | 30.74 | 3.22 | 0.79 | 1.84 | 0.05 |
| 5 hours | 30.4 | 2.81 | 0.83 | 1.14 | 0.19 |
| 12 hours | 24.07 | 6.52 | 0.93 | 0.24 | 2.4E-6 |

**Table S1. Second exposure fitting parameters**

Parameters describing the repair kinetics of the different radiation exposure modalities as obtained by Equation (4)

**Figure S1. Background level of γ-H2AX after mock irradiation.**

Number of γ-H2AX foci in AG01522 cells exposed to mock irradiation. Time 0 represents the moment of the (mock) irradiation. The data are obtained after 3 independent experiments and the error bars represent the standard error of the mean.

**Figure S2. Co-localization of γ-H2AX and 53BP1 foci after single and split irradiations (fixed after 30 minutes after irradiation).**

γ-H2AX and 53BP1 pictures taken after 30 minutes of irradiation for: (first row) Single Dose exposure, (second row) Split dose with a time interval of 1 hour. The images are taken 30 minutes after the 2^nd^ exposure. (third row) Split dose with a time interval of 6 hour. The images are taken 30 minutes after the 2^nd^ exposure

**Figure S3. Co-localization of γ-H2AX and 53BP1 foci after single and split irradiations (fixed after 1 hour after irradiation)**

γ-H2AX and 53BP1 pictures taken after 30 minutes of irradiation for: (first row) Single Dose exposure, (second row) Split dose with a time interval of 1 hour. The images are taken 30 minutes after the 2^nd^ exposure. (third row) Split dose with a time interval of 6 hour. The images are taken 30 minutes after the 2^nd^ exposure
